# Evaluation of Pm2.5 Influence on Human Lung Cancer Cells Using a Microfluidic Platform

**DOI:** 10.7150/ijms.94803

**Published:** 2024-04-22

**Authors:** Uyen Thi Nhat Nguyen, Han-Yun Hsieh, Tzu-Yun Chin, Guani Wu, Yen Po Lin, Ching-Yi Lee, Yi-Chiung Hsu, Yu-Jui Fan

**Affiliations:** 1 International Ph.D. Program in Cell Therapy and Regenerative Medicine, Taipei Medical University, 250 Wuxing St., Taipei 11031, Taiwan.; 2 Institute of Applied Mechanics, National Taiwan University, Taipei 10617 Taiwan.; 3 Department of Life Science, National Dong-Hwa University, Hualien, 974301 Taiwan.; 4 Department of Biomedical Sciences and Engineering, National Central University, Taoyuan 320, Taiwan.; 5 Department of Statistics & Data Science, University of California Los Angeles, Los Angeles, CA 90095, USA.; 6 Department of Critical Care Medicine, Taipei Tzu Chi Hospital, Buddhist Tzu Chi Medical Foundation, New Taipei 231, Taiwan.; 7 The Division of Chest Medicine, Department of Internal medicine, Taoyuan General Hospital, Tao Yuan, Ministry of Healthy and Welfare, Taoyuan 330, Taiwan.; 8 Center for Astronautical Physics and Engineering, National Central University, Taoyuan, 320, Taiwan.; 9 School of Biomedical Engineering, Taipei Medical University, 250 Wuxing St., International PhD Program for Biomedical Engineering, Taipei 11031, Taiwan.

**Keywords:** Lung cancer, PM2.5, invasion, migration, ROS, mitochondrial dysfunction

## Abstract

In this study, we developed a microfluidic device that is able to monitor cell biology under continuous PM2.5 treatment. The effects of PM2.5 on human alveolar basal epithelial cells, A549 cells, and uncovered several significant findings were investigated. The results showed that PM2.5 exposure did not lead to a notable decrease in cell viability, indicating that PM2.5 did not cause cellular injury or death. However, the study found that PM2.5 exposure increased the invasion and migration abilities of A549 cells, suggesting that PM2.5 might promote cell invasiveness. Results of RNA sequencing revealed 423 genes that displayed significant differential expression in response to PM2.5 exposure, with a particular focus on pathways associated with the generation of reactive oxygen species (ROS) and mitochondrial dysfunction. Real-time detection demonstrated an increase in ROS production in A549 cells after exposure to PM2.5. JC1 assay, which indicated a loss of mitochondrial membrane potential (ΔΨm) in A549 cells exposed to PM2.5. The disruption of mitochondrial membrane potential further supports the detrimental effects of PM2.5 on A549 cells. These findings highlight several adverse effects of PM2.5 on A549 cells, including enhanced invasion and migration capabilities, altered gene expression related to ROS pathways, increased ROS production and disruption of mitochondrial membrane potential. These findings contribute to our understanding of the potential mechanisms through which PM2.5 can impact cellular function and health.

## Introduction

Particulate matter (PM), as known a key type of air pollutant, has been regarded as a group 1 human carcinogen by the International Agency for Research on Cancer (IARC) 1. Hefty pollutants produced from factory emissions, vehicle emissions, electricity production, and firecracker burning releasing poisonous heavy metals and dioxin may generate particulate matter (PM) with an aerodynamic diameter of 2.5 µm or less (PM2.5) 2. There are two kinds of atmospheric particulate matter. One is particulate matter directly emitted from coal plants called primary PM, and the other is formed from the conversion of SO_2_ and NO_2_ emissions into particulates of sulfate and nitrates called secondary PM 3. PM2.5 is considered not only harmful to human health 1,4,5, but also exacerbates the *risk factors* of many diseases, such as hypertension, stroke, inflammation, asthma, pulmonary diseases, and cardiovascular diseases 6-11. Furthermore, a long-term exposure study collected cohort data by the American Cancer Society 12 shows each 10-µg/m^3^ increase of exposure to PM2.5 affects an approximately 8% increase in risk of lung cancer; survival was significantly affected by PM2.5 13. Also, metal components and ions of PM2.5 binding to the surface of PM2.5 particles may be responsible for systemic inflammation 14. Recent reports indicated that induced oxidative stress caused by PM2.5 exposure was strongly linked to lung cancer 15-18 by reactive oxygen species (ROS) is a production from an imbalance of oxidative stress, and is involved in cell signaling proteins (such as Nuclear factor kappa B (NF- κB), Tumor necrosis factor alpha (TNF-α), mitogen-activated protein kinases (MAPKs)), and cytokines (Interleukins (ILs)) that play a role in the development or progression of pulmonary diseases 17,19. From that, many biological pathways regulated by PM2.5 exposure could cause existing pulmonary diseases over the short or long term 18,20-23. PM2.5 with its small size allows better tissue penetration, which is related to their uptake in epithelial cells, and also to their ability to induce oxidative stress through inducing cellular heme oxygenase-1 (HO-1) expression and depleting intracellular glutathione is associated with damage in the mitochondria 24 or activation of protein-1 and NF-κB signaling pathways that are involved in allergic inflammation 25. Another study shows exposure to PM2.5 in 12 hours and 24 hours could upregulating ROS-dependent NOD-like receptors pyrin domain containing 3 (NLRP3) inflammasome-mediated pyroptosis, and cell cycle arrest in the G2 phase in the RAW264.7 cells that all induced lung damage (18). The new finding declared epidermal growth factor receptor (EGFR) pathway was activated in lung cancer cells through short-term exposure to PM2.5 for 24 hours while long-term exposure in 90 days could enhance the transmembrane serine protease 2-IL18 (TMPRSS2-IL18) pathway that promotes tumor progression in lung cancer 26.

To date, ROS-detecting techniques can observe at the endpoint and qualitatively, but have difficulties identifying the amount of ROS production and continuous measurement 27,28. As a result, microfluidic techniques may be a new hope for detecting sensitivity of ROS on toxicity mechanisms 27,29,30. Many studies used microfluidic or lab-on-a-chip devices as single-cell analytical tools that help to increase sensitivity and minimize dilution effects 31 and apply a high biological for inflammatory treatment 30,32 and illuminate the pathophysiology or screening 33,34. Moreover, biological network-based systems toxicology models have been used in preclinical tobacco studies that show metabolism biomarkers and signaling associated with respiratory or cardiovascular diseases 35. Besides, the developed next-generation sequencing (NGS) technique provides a tool for investigating toxicological effects through gene expression from cell/tissue exposure to chemicals but limited toxicological information.

Although the mechanism of toxicity of PM2.5 to lung cancer has been studied widely but remains to be unclear all. It is still a major challenge to identify biological targets in the human gene regulation network. To contribute to clarifying these questions, in this study, we investigated alterations in the protein profile and gene expression profile of adenocarcinoma human alveolar basal epithelial cell (A549 cells) treated with PM2.5 using mass spectrometry (MS) and next-generation sequencing (NGS). Thus, PM2.5 alters the proteomic and transcriptome of genes might be relevant for PM2.5-induced lung cancer.

## Material and methods

### Material

A549 cells were purchased from ATCC (USA). PM2.5 and Fetal bovine serum (FBS) were purchased from Sigma-Aldrich (USA), and penicillin/streptomycin was from Thermo Fisher Scientific (USA), while prestoBlue and TRIzol reagent were purchased from Invitrogen (USA). DMEM/F12 medium was provided by Hyclone (USA). RNA 6000 labchip kit and Agilent SureSelect Strand Specific RNA Library Preparation Kit were obtained from BAgilent technologies (USA). Others, such as Muse™ Annexin V, Dead Cell Kit, and Muse MitoPotential, were from Luminex (USA), while the JC1 kit was from eLabscience (USA), and Matrigel was from Corning (USA).

### Preparation and exposure

Diesel particulate matter NIST 1650b (PM2.5) suspended in dimethyl sulfoxide (DMSO) to a final concentration of 25 mg/ml and sonicated for 30 minutes. The stock solution was stored at room temperature. Then dilute with culture medium to experimental concentration.

### MTT assay

6x10^3^ / 100 µl cells were seeded in each 96 well and incubated 37°C for 24hr. Then, culture medium was exchanged with fresh medium containing PM2.5 at various concentrations (0, 25, 50, 75, 100 µg/ml) and no PM2.5 treated group was set as a control group. After incubator time (0hr, 24hr, and 48hr), solution was removed, then 100 μl MTT reagent (Invitrogen) was added for 3 hours. After MTT reagent was removed, 100 μl DMSO was added to dissolve. The absorbance value was measured with wavelength 550 nm by ELISA reader to analysis of proliferation. For in all other assays, cells were exposed with PM2.5 at 50 μg/ml concentration.

### Cell viability

1x10^4^ / 100 µl cells were seeded in each 96 well and incubated 37°C. After cells attached, 100 µl PM2.5 solution (50 µg/ml) was added for 24 and 48 hours. After solution was removed, 100 μl PrestoBlue reagent (Invitrogen) was added for 3 hours. The absorbance value was measured with wavelength 570 nm by ELISA reader.

### Invasion assay

Matrigel (Corning) was coated in upper chamber of the transwell plate for 1.5 hours in 37°C incubator. After matrigel solidification, 2x10^4^/200 µl cells containing serum-free medium was seeded in upper chamber. 700 µl medium containing FBS was added in well and upper chamber was putted in well at 37°C for 18 hours. Finally, the cells were stained with crystal violet and counted.

### Migration assay

3x10^4^/100 µl cells were seeding in culture-insert. After cells attached, culture-insert was removed and pictures were taken at 0^th^ hour, 12^th^ hour, 24^th^ hour. Then the migration area was quantified by Image J.

### RNA extraction

RNA extraction was performed by employing TRIzol reagent (Invitrogen) to isolate total RNA samples. The extracted RNA was then evaluated for both quantity and purity using a BioDrop instrument. To ensure accurate assessment, agarose electrophoresis was utilized for qualitative analysis, while a Bioanalyzer 2100 (Agilent Technology, USA) with the RNA 6000 labchip kit (Agilent Technologies, USA) was employed to verify the quality of the RNA.

### Microfluidic device fabrication

The microfluidic channel's inlet and outlet were connected to a syringe filled with medium, and a syringe pump was utilized to maintain a continuous flow of fluid within the microfluidic channel. The microfluidic channel contained 2 parts, bottom glasses (width 25.4 mm, length 38 mm, height 1 mm) and Polydimethylsiloxane (PDMS) microfluidic channel (width 1 mm, length 18 mm, height 82 µm). PDMS was employed in the construction of a microfluidic channel, this material exhibiting features such as transparency to light, excellent biocompatibility, and high elasticity.

### Library preparation & sequencing

RNA libraries were generated using the Agilent SureSelect Strand Specific RNA Library Preparation Kit. The resulting libraries were sequenced on the illumina Novaseq6000 platform, employing 150 paired-end reads. As per Illumina's standard sequencing protocol, it was anticipated that the raw sequences would yield approximately 20 million reads per sample.

### RNA-seq analysis

The generated sequences underwent a filtering procedure to extract high-quality reads. Reads with low quality scores, as well as those containing adapters or ploy-N sequences, were trimmed or removed. The filtered reads were then aligned to the reference genomes using Bowtie2 (version 2.3.4.1). After filtering out low-quality data, the remaining qualified reads were subjected to gene expression estimation using RSEM. The gene expression levels were calculated as TPM (Transcripts Per Million). For differential expression analysis, statistical analyses of the gene expression profiles were performed using edgeR v3.5. The reference genome and gene annotations used in the analysis were obtained from the Ensembl database. Pathway enrichment illustrating the molecular relationships between genes were generated using the Ingenuity Pathway Analysis software (https://apps.ingenuity.com). The Core Analysis feature of the software was utilized to highlight associations that were found to be statistically significant (P < 0.05).

### ROS detection

A549 cells were seeded at a density of 1x10^6^ cells/ml with a ROS real-time indicator (LumiSTAR, Taiwan) inside a PDMS microfluidic channel for 24 hours. PM2.5 particles (50 μg/ml) were then added to the culture medium and injected into the microfluidic chamber under a flow rate of 7 μl/s. Fluorescent images were taken every 30 minutes using an immunofluorescence microscope (Olympus, Japan) and analyzed using the HCImages software. DCFDA assay (Abcam, USA) also performed the ROS accumulation after 24 hours PM2.5 administration, according to the manufacture's instruction. After administrated with PM2.5, cells were stained with DCFDA for 45 minutes, and observed with confocal microscope.

### Mitochondrial membrane potential assay

The Muse MitoPotential assay (Luminex, USA) was used to measure mitochondrial health and cell death. A549 cells were seeded at a density of 1x10^4^ cells/ml in a 6-well culture plate and PM2.5 solution (50 µg/ml) was added for 24 hrs. Following the instruction, the working solution was added to each tube and incubated for 20 minutes at 37°C. Then, the Muse MitoPotential reagent was added to each tube and incubated for 5 minutes at room temperature. Finally, samples were analyzed using the Muse Cell Analyzer.

### JC1 assay

The decrease of mitochondrial membrane potential is a significant event that serves as an indicator during the initial stages of apoptosis. A widely employed fluorescent probe called JC-1 is utilized for detecting changes in mitochondrial membrane potential (∆Ψm). The relative ratio of red and green fluorescence is commonly used to measure the degree of mitochondrial depolarization (eLabscience, USA). The maximum excitation wavelength for the JC-1 monomer is 514 nm, with a corresponding maximum emission wavelength of 529 nm. On the other hand, the JC-1 polymer has a maximum excitation wavelength of 585 nm and a maximum emission wavelength of 590 nm. Red and green fluorescence were observed with confocal microscope, the relative ratio of red and green fluorescence was calculated by Image J software.

### Quantification and statistical analysis

Three replicates were designed in each group and each assay. The data is reported as mean± standard error (SEM). P values were calculated using two statistical methods: Two-sided paired T-tests or One-way Analysis of Variance (ANOVA) with GraphPad Prism 9 (*P <0.05, **P <0.01, *** P <0.001).

## Results

### Cell viability effect

**Figure 1:**
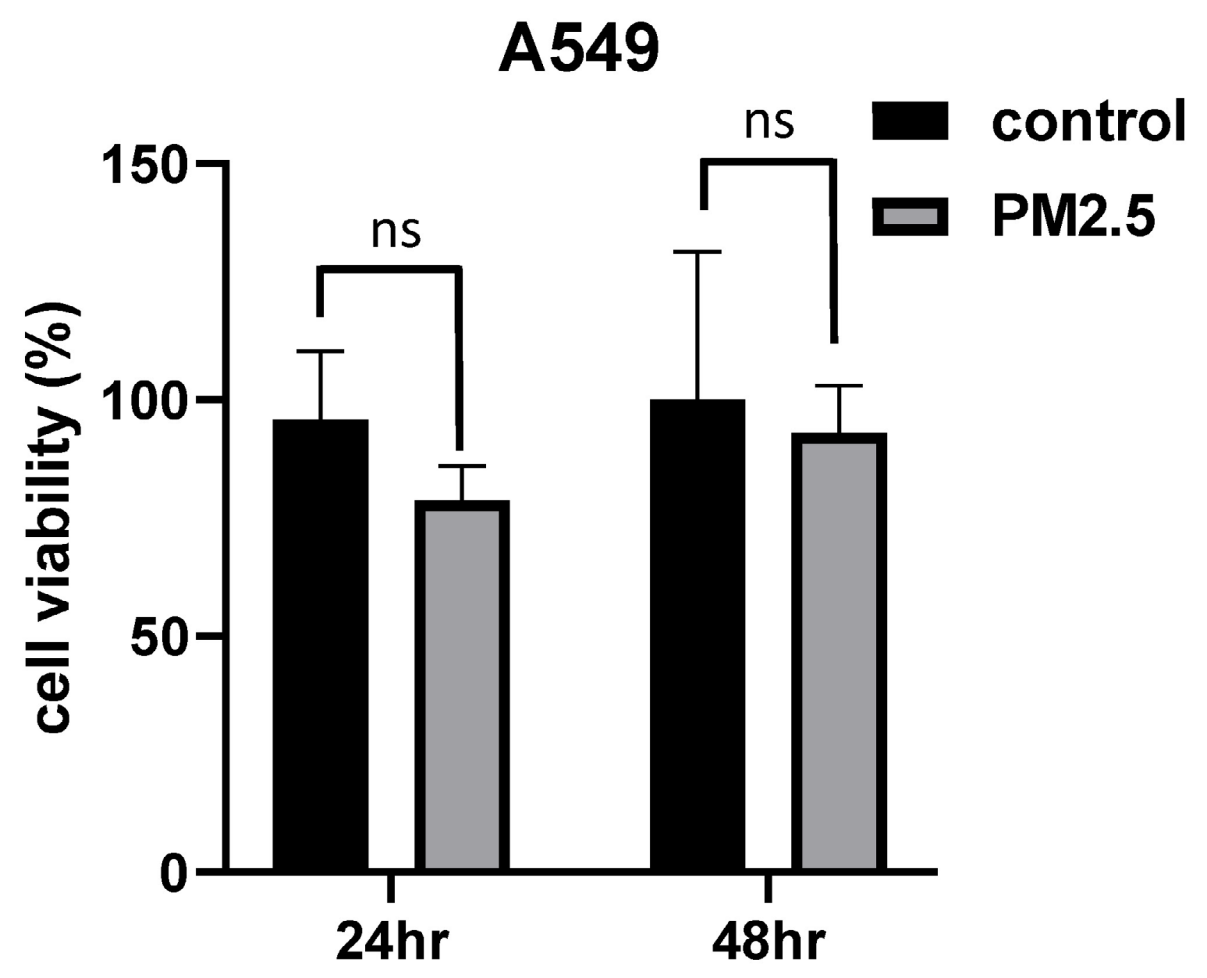
The histograms show cell viability measured by MTT assay after cells exposed to PM2.5 for 24 hours and 48 hours.

To evaluate the potential toxicity of PM2.5 on A549 cells, we conducted exposure experiments for durations of 24 and 48 hours. During this study, the A549 cells were exposed to PM2.5 particles (50 μg/ml) for both periods, as were presented in another study of PM2.5 promotes lung cancer progression 26. Surprisingly, the experimental results did not reveal any statistically significant differences in cell viability between the cells exposed to PM2.5 and the control group. This suggests that, based on our assessment of cell viability, the exposure to in the specific experimental conditions we employed, it was observed that the exposure of A549 cells to PM2.5 did not lead to cellular injury to cell death (Figure 1).

### PM2.5 promotes A549 invasion and migration

**Figure 2:**
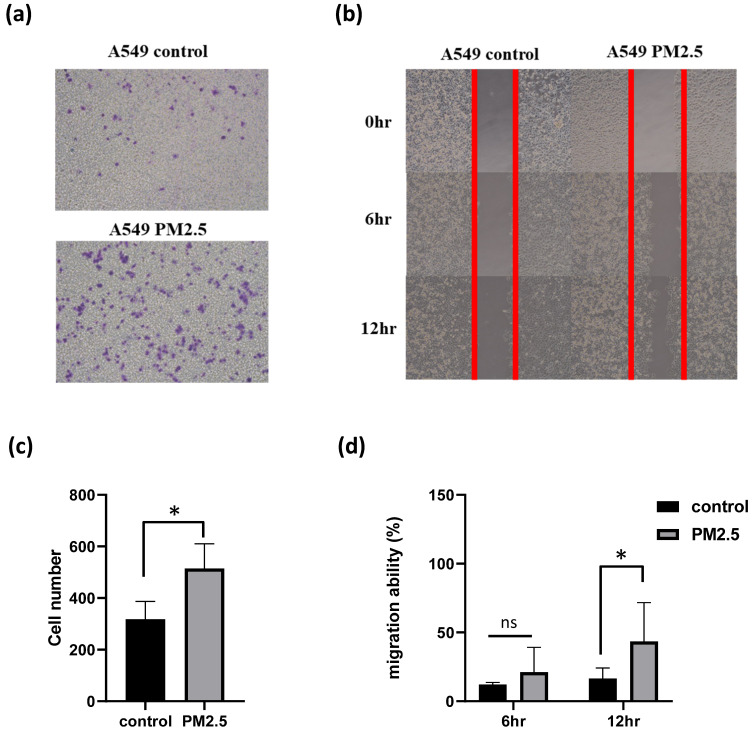
Effects of PM2.5 on migration and invasion in A549 cells. (a), (c) Invasion assay was used to assess the effect of PM2.5 on A549 invasion at 18 hours after PM2.5 treatment. (b), (d) Scratch wound assay was used to assess the effect of PM2.5 on A549 migration at 6 hours and 12 hours after PM2.5 treatment.

To assess the impact of PM2.5 exposure on the invasive and migratory properties of A549 cells, we conducted invasion and migration assays. Notably, the experimental findings unveiled an intriguing outcome, indicating that PM2.5 exposure can potentially enhance the invasion ability of A549 cells (Figure 2a, c). In the invasion assay, where we evaluated the invasive behavior of the cells, we observed a notable increase in the invasiveness of A549 cells upon exposure to PM2.5. Moreover, in the migration test, we quantified the migratory behavior of the cells using Image J software, and the results demonstrated a significant elevation in the migration ability of A549 cells following exposure to PM2.5 (Figure 2b, d). These observations underscore the potential influence of PM2.5 on the invasive and migratory properties of A549 cells and warrant further investigation to unravel the underlying mechanisms involved.

### Gene expression profile of cells exposed to PM2.5

**Figure 3:**
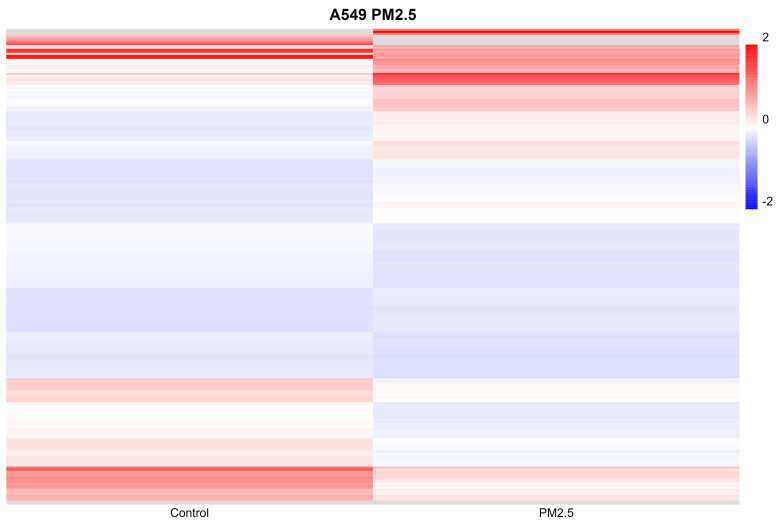
Differential expression gene profile of cells exposed to PM2.5. In order to determine which genes make the difference between cells exposed to PM2.5, NGS was performed on cells exposed to PM2.5 for 24 hours*.*

To investigate the impact of PM2.5 exposures on gene regulation, we conducted RNA sequencing (RNAseq) analysis on cells subjected to both conditions for a duration of 24 hours. Genes exhibiting a fold change greater than 2 or less than -2 were considered statistically significant. In the A549 cell line, we identified a total of 423 genes that displayed significant differential expression (Figure 3). Following the identification of 423 genes exhibiting significant differential expression in the A549 cell line, we proceeded to perform functional annotations using Ingenuity Pathway Analysis (IPA) to gain insights into the biological implications of these genes.

### Functional annotations of the differentially expressed genes

**Figure 4:**
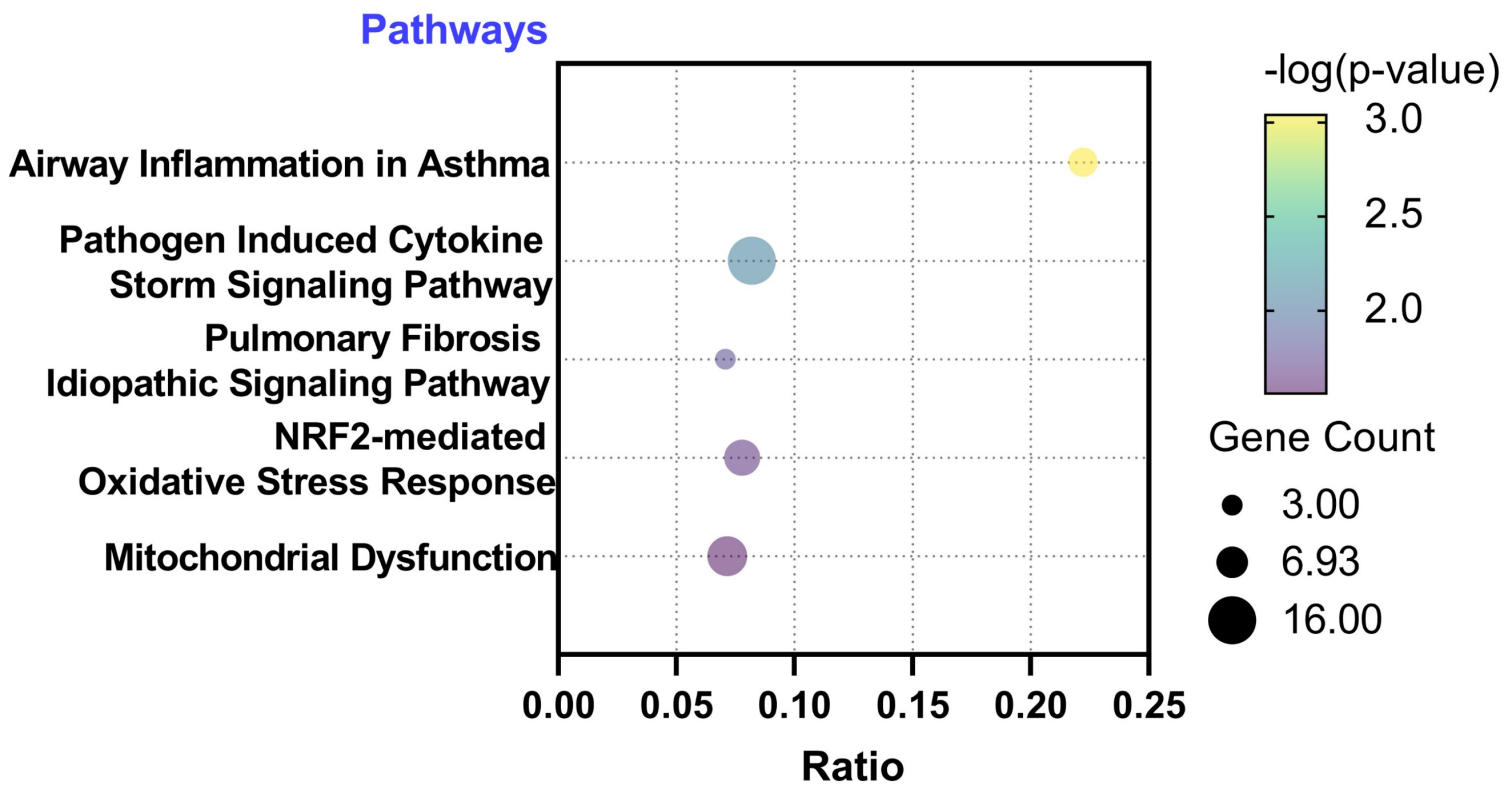
Ingenuity Pathway Analysis (IPA) with differentially expressed genes. IPA identified signaling pathways that exhibited a significant association (P-value < 0.05) with the differentially expressed genes.

Subsequently, we conducted functional annotations of these differentially expressed genes using Ingenuity Pathway Analysis (IPA). Through this analysis, we identified signaling pathways that exhibited a significant association (*P*-value < 0.05) with the differentially expressed genes. Remarkably, we observed a strong correlation between the 423 significantly different genes in the A549 cell line and pathways related to ROS generation. Among the top five significant pathways identified in A549, the following were noteworthy: Pathogen Induced Cytokine Storm Signaling Pathway (p = 9.12E-4), Pulmonary Fibrosis Idiopathic Signaling Pathway (p = 7.94E-3), Airway Inflammation in Asthma (p = 1.6E-2), Nuclear factor erythroid 2-related factor 2 (NRF2)-mediated Oxidative Stress Response (p = 2.1E-2), and Mitochondrial Dysfunction (p = 2.7E-2). These findings highlight the potential involvement of these pathways in the response of A549 cells to PM2.5 exposure (Figure 4). Through IPA analysis, we aimed to unravel the signaling pathways that showed a substantial association with the differentially expressed genes. Notably, our analysis revealed a strong correlation between the significantly different genes and pathways involved in the generation of ROS. These findings provide valuable information about the potential molecular mechanisms underlying the impact of PM2.5 exposure on gene regulation in the A549 cells.

### Administration of PM2.5 increased ROS production under real-time detection

**Figure 5:**
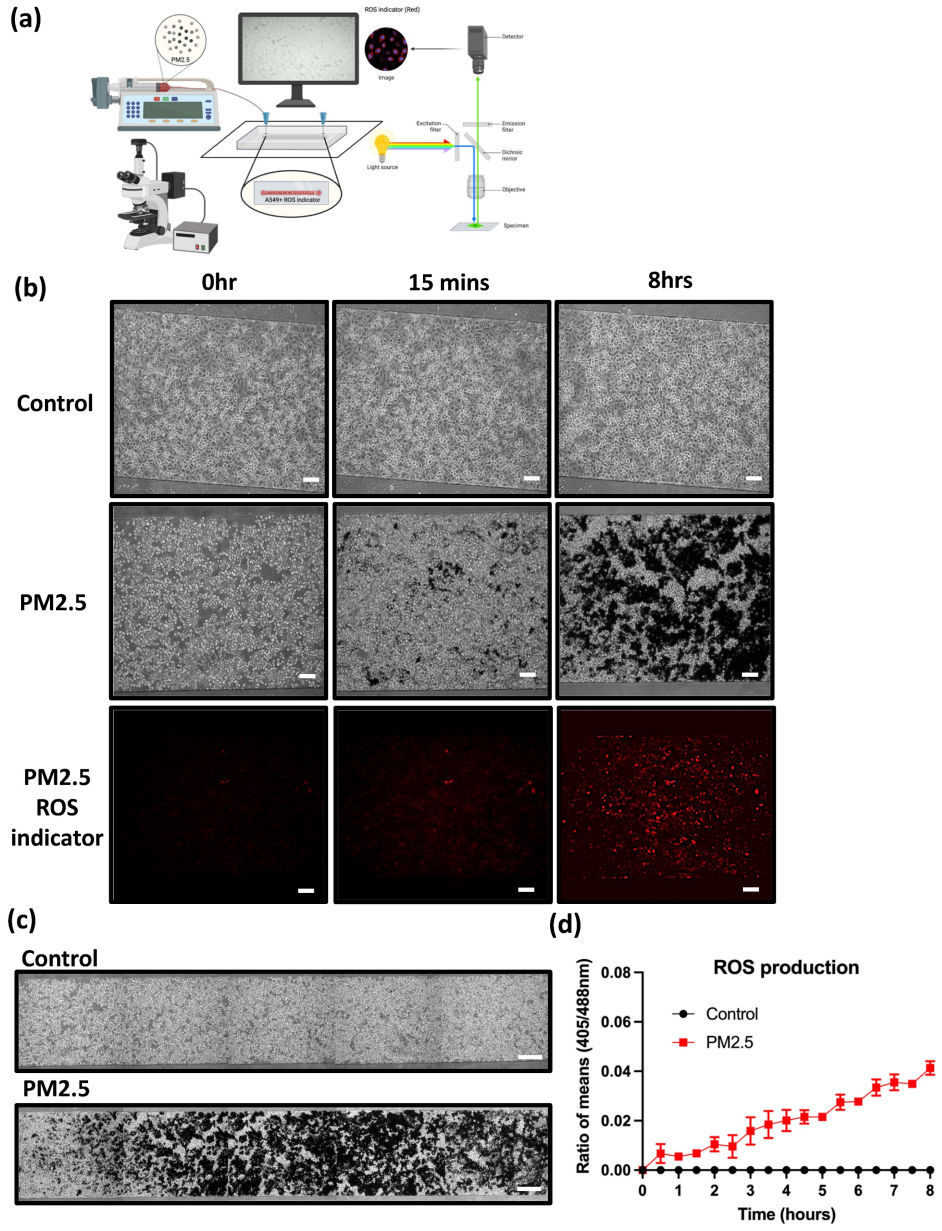
Observation of A549 real-time ROS production under PM2.5 treatment. (a) Microfluidic channel with real-time detection device. (b) Cell morphology and ROS production under different time points. (c) Statistics of real-time ROS production. Scale Bar: 100 µm.

In this research, we aim to investigate whether PM2.5 administration increases ROS production. The experimental setup, illustrated in Figure 5(a), comprised a syringe pump and a stage top incubator for culturing cells on a microscope.

A549 cells were seeded inside a microfluidic channel and observed using an immunofluorescence microscope for ROS production. Under the flow rate of 40 µl/min, Figure 5 (b) shows images at different time points. PM2.5 levels slightly increased after 15 minutes of PM2.5 exposure and reached saturation after 8 hours treatment.

**Figure 6:**
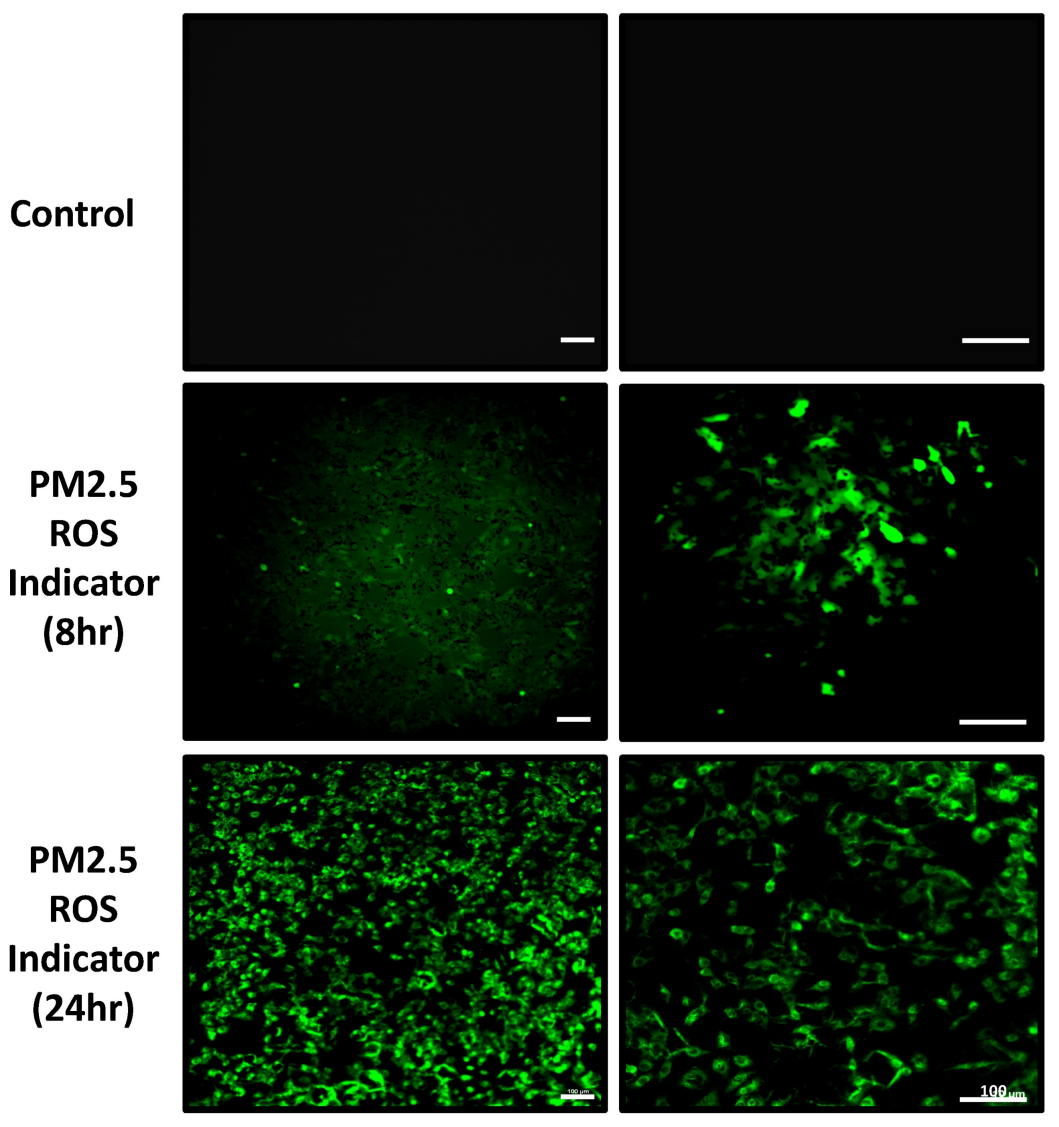
Observation of A549 ROS production under PM2.5 treatment. The immunofluorescence signal of ROS was observed with confocal microscope. Scale Bar: 100 µm.

In addition to real-time ROS detection, we also analyzed the ROS accumulation after 24-hour PM2.5 administration. The data indicates that after 24 hours of incubation with PM2.5, ROS levels significantly increased compared to the control group (Figure 6).

### JC1 assay and mitochondrial membrane potential assay of A549 cells under PM2.5 administration

**Figure 7:**
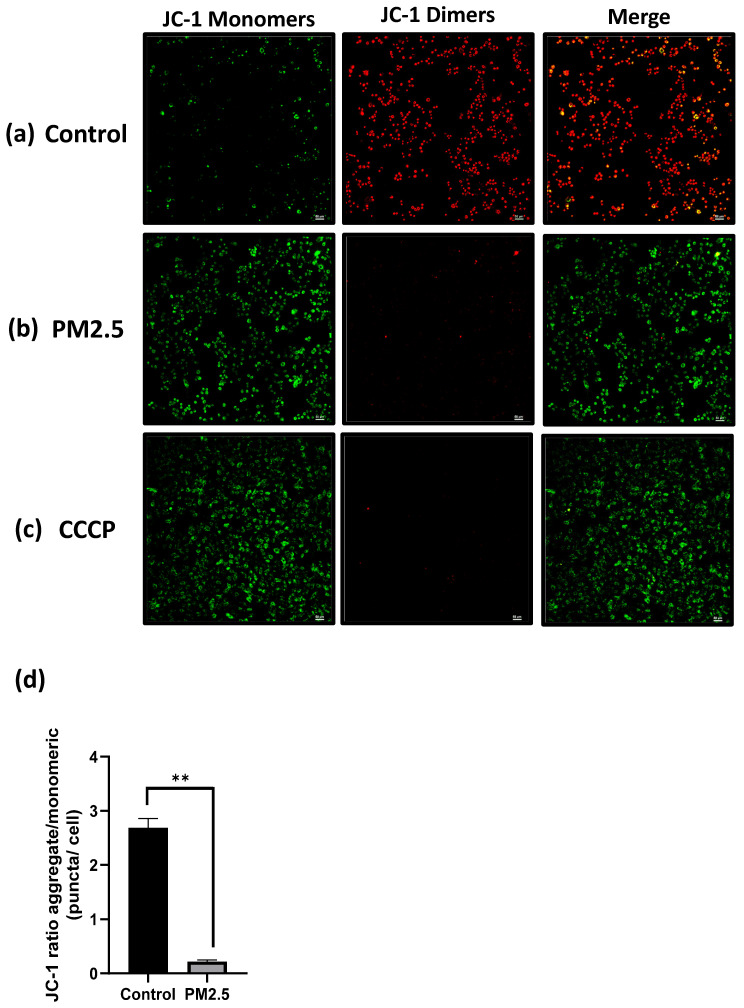
JC1 assay. Mitochondria membrane potential (ΔΨm) detection under PM2.5 treatment. Scale Bar: 50µm. (a) Confocal images. Green: JC-1 monomers, Red: JC-1 dimers. Scale Bar: 50 µm (b) Statistics. Each experiment was performed in triplicate.

The effect of PM2.5 on mitochondrial membrane potential (MMP or ΔΨm) was assessed using the JC-1 assay and flow cytometry. In JC-1 assay, which measures the fluorescence ratio transitioning from red to green. This transition was clearly observable in the fluorescence images and quantified using ImageJ software. Following a 24-hour PM2.5 flowing in microfluidic channel or the CCCP group (positive control), A549 cells exhibited primarily green fluorescence from JC-1 monomers, with minimal red fluorescence, as illustrated in Figure 7 (a), (b), (c). In contrast, the control group displayed the formation of JC-1 dimers within the mitochondrial matrix, resulting in intense red fluorescence (Figure 7a).

**Figure 8:**
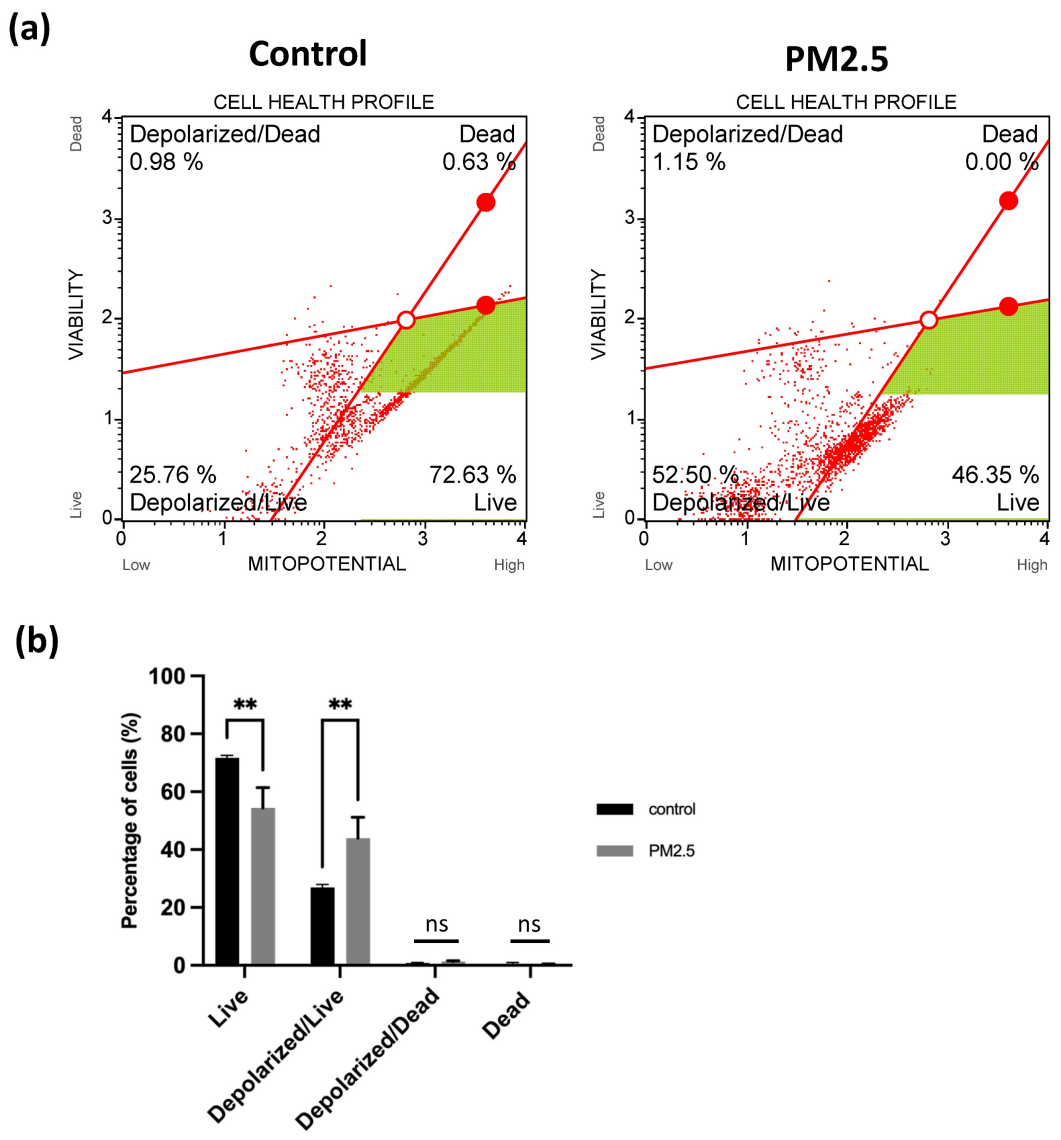
Observation of A549 mitochondria membrane potential under PM2.5 treatment. (a) Flow Cytometry analysis. (b) Statistics. Each experiment was performed in triplicate.

Figure 7 (d) depicts the red/green fluorescence ratio, which was 2.69 ± 0.097 for the control group and 0.22187 ± 0.015 for the PM2.5 group. In the flow cytometry analysis, live cell was decreased from 72.63% to 46.35% (Figure 8a), Depolarized cell also had 2-fold increase compared to control group (Control: 25.76%, PM2.5: 52.5%). These findings indicate that PM2.5 can penetrate A549 cells, leading to depolarization and the subsequent loss of mitochondrial membrane potential (ΔΨm).

## Discussion

In this study, we investigated the effects of PM2.5 exposure on A549 cells, a type of human lung cancer epithelial cells. The results indicated that PM2.5 promoted the invasion and migration of these cells. Through RNA sequencing analysis, we identified 423 genes that exhibited significant differential expression in response to PM2.5 exposure. Functional annotations using Ingenuity Pathway Analysis (IPA) revealed a strong correlation between these differentially expressed genes and pathways associated with reactive oxygen species (ROS) generation. Notably, pathways such as Pathogen Induced Cytokine Storm Signaling, Pulmonary Fibrosis Idiopathic Signaling, Airway Inflammation in Asthma, NRF2-mediated Oxidative Stress Response, and Mitochondrial Dysfunction were significantly associated with the differentially expressed genes. Furthermore, this study demonstrated that PM2.5 exposure increased ROS production in A549 cells, with levels significantly higher after 24 hours of incubation compared to the control group. Flow cytometry analysis revealed a significant reduction in the number of live cells exposed to PM2.5 compared to the control group. About the mitochondrial dysfunction, JC-1 assay was performed. The control group displayed intense red fluorescence, while the PM2.5 group exhibited predominantly green fluorescence. This further supports the notion that PM2.5 exposure leads to depolarization and subsequent loss of mitochondrial membrane potential in A549 cells, and also provide valuable insights into the molecular mechanisms underlying the impact of PM2.5 exposure on gene regulation in lung cells, emphasizing the potential health risks associated with air pollution.

The study aimed to investigate the effects of PM2.5 on A549 cells and develop a microfluidic device for real-time detection of ROS variations. Our preliminary experiments revealed that PM2.5 exposure did not significantly decrease cell viability, indicating the absence of cellular injury or death. Similarly, previous studies have demonstrated that A549 cell lines still keep their ability to proliferate under PM2.5-short exposed 22,26,36,37. Besides, another study indicated PM2.5 promotes to migrate and invasion under PM2.5-exposed 26,36 through the altering of related mechanisms. In our studies, PM2.5 exposure enhanced invasion and migration capabilities, altered gene expression associated with ROS pathways, increased ROS production and disrupted mitochondrial membrane potential. This agrees with the recent studies; PM2.5 can cause oxidative stress 38,39, cytotoxicity 40,41, and affect the up or down-regulation of the release of mediators of the signaling pathway involved 41,42. These findings significantly contribute to understanding of how PM2.5 impacts cellular function and health and have implications for environmental health research and potential intervention strategies. Additionally, the study explored the potential of microfluidic techniques for in vitro toxicity studies of PM2.5 exposure, highlighting their value as an alternative to conventional assay formats.

### Limitations of the study

Overall, the findings highlight the potential adverse effects of PM2.5 on A549 cells, including enhanced invasion and migration, altered gene expression, increased ROS production and disruption of mitochondrial membrane potential. This provides valuable insights into the molecular mechanisms underlying the impact of PM2.5 exposure on gene regulation in lung cells, emphasizing the potential health risks associated with air pollution.
